# Inhibition of the mitochondrial calcium uniporter rescues dopaminergic neurons in *pink1* ^*−*/*−*^ zebrafish

**DOI:** 10.1111/ejn.13473

**Published:** 2016-12-28

**Authors:** Smijin Soman, Marcus Keatinge, Mahsa Moein, Marc Da Costa, Heather Mortiboys, Alexander Skupin, Sreedevi Sugunan, Michal Bazala, Jacek Kuznicki, Oliver Bandmann

**Affiliations:** ^1^ International Institute of Molecular and Cell BiologyWarsawPoland; ^2^ Bateson CentreUniversity of SheffieldSheffieldUK; ^3^ Sheffield Institute for Translational Neuroscience (SITraN)University of Sheffield385a Glossop RoadSheffieldS10 2HQUK; ^4^ Luxembourg Centre for Systems BiomedicineUniversity of LuxembourgLuxembourgLuxembourg; ^5^ National Centre for Microscopy and Imaging ResearchUniversity of California San DiegoLa JollaCAUSA

**Keywords:** *Danio rerio*, MICU1, Parkinson's disease, Ruthenium red

## Abstract

Mutations in *PTEN‐induced putative kinase 1* (*PINK1*) are a cause of early onset Parkinson's disease (PD). Loss of PINK1 function causes dysregulation of mitochondrial calcium homeostasis, resulting in mitochondrial dysfunction and neuronal cell death. We report that both genetic and pharmacological inactivation of the mitochondrial calcium uniporter (MCU), located in the inner mitochondrial membrane, prevents dopaminergic neuronal cell loss in *pink1* ^Y431^* mutant zebrafish (*Danio rerio*) via rescue of mitochondrial respiratory chain function. In contrast, genetic inactivation of the voltage dependent anion channel 1 (VDAC1), located in the outer mitochondrial membrane, did not rescue dopaminergic neurons in PINK1 deficient *D. rerio*. Subsequent gene expression studies revealed specific upregulation of the *mcu* regulator *micu1* in *pink1* ^Y431^* mutant zebrafish larvae and inactivation of micu1 also results in rescue of dopaminergic neurons. The functional consequences of PINK1 deficiency and modified MCU activity were confirmed using a dynamic *in silico* model of Ca^2+^ triggered mitochondrial activity. Our data suggest modulation of MCU‐mediated mitochondrial calcium homeostasis as a possible neuroprotective strategy in PINK1 mutant PD.

## Introduction

Parkinson's disease (PD) is a common neurodegenerative disorder, resulting in both motor and non‐motor symptoms (Kalia & Lang, 2015). The pathological hallmark of PD is loss of dopaminergic neurons in the substantia nigra pars compacta. Mitochondrial dysfunction was first described in post mortem tissue of patients with sporadic PD. More recently, it has been closely linked to Mendelian forms of familial PD, in particular to early onset PD due to *parkin*, PTEN‐induced putative kinase 1 (*PINK1*) and *DJ‐1* mutations (Exner *et al*., 2012). Loss of PINK1 function results in mitochondrial calcium overload, thought to be due to impaired calcium efflux from the mitochondria (Gandhi *et al*., 2009; Marongiu *et al*., 2009). The reduced mitochondrial calcium capacity in PINK1 deficient neurons results in increased oxidative stress which in turn leads to reduced glucose uptake and a lowering of the threshold for the opening of the permeability transition pore (PTP). This suggests that impaired mitochondrial calcium homeostasis is at least one of the pathological mechanisms resulting from PINK1 deficiency.

Mitochondria are key modulators of intracellular calcium homeostasis. The voltage‐dependent anion channel 1 (VDAC1) is located within the outer mitochondrial membrane and transports calcium into the intermembrane space (Shoshan‐Barmatz & Golan, 2012). Calcium then enters the mitochondrial matrix through a dedicated channel within the inner mitochondrial membrane referred to as the mitochondrial calcium uniporter (MCU) (Baughman *et al*., 2011; De Stefani *et al*., 2011). Silencing MCU severely reduces mitochondrial calcium uptake but mitochondrial respiration and membrane potential remain fully intact. The MCU channel is part of a multisubunit modular complex that includes regulators of its activity, calcium sensitivity and expression (Murgia & Rizzuto, 2015). Additional components of this four MCU complex include mitochondrial calcium uptake 1 and 2 (MICU1, MICU2), the essential MCU regulator (EMRE) and the mitochondrial calcium uniporter regulator 1 (MCUR1) (see Murgia & Rizzuto, 2015 for review). Recently, a physical and functional interaction of MCU with VDAC1 has also been reported (Liao *et al*., 2015).

Zebrafish are increasingly used to study mechanisms linked to human neurodegenerative diseases. They are vertebrates and therefore more closely related to humans than *Caenorhabditis elegans* or *Drosophila*. Their genome is fully characterized and contains orthologues for approximately 70% of all human genes, but 82% for all human disease genes (Howe *et al*., 2013). We recently reported our findings in a zebrafish mutant line carrying a Stop mutation (*pink1* ^Y431^*, from here on referred to as *pink1* ^*−*/*−*^ for homozygous *pink1* mutant larvae) in the kinase domain of *pink1*, the zebrafish orthologue of the human PD gene *PINK1* (Flinn *et al*., 2013). *pink1* ^−/−^ already resulted in impaired function of the mitochondrial respiratory chain and loss of dopaminergic neurons at 3 days post fertilization (dpf). Zebrafish embryos are eminently amenable to both genetic and pharmacological manipulation.

Here, we demonstrate a rescue effect of both pharmacological and genetic MCU inhibition on the dopaminergic neurons via normalisation of mitochondrial function. We further report specific transcriptional upregulation of *micu1* in *pink1* ^−/−^ zebrafish larvae. Genetic inactivation of *micu1* also results in rescue of the dopaminergic neurons. In contrast, genetic inactivation of *vdac1* did not protect the dopaminergic neurons in *pink1* ^−/−^ zebrafish larvae.

To understand these findings on a more mechanistic level, we applied our previously developed mathematical model to investigate the role of modified Ca^2+^ dynamics on the respiratory function (Komin *et al*., 2015). Incorporating the reported effects of PINK1 on Ca^2+^ homeostasis (Gandhi *et al*., 2009; Gautier *et al*., 2012) into the model reveals a dramatic decrease in respiratory capacity that can be compensated by modified MCU activity. This independent confirmation of the experimental results strongly supports the essential role of mitochondrial energy metabolism to dopaminergic neuronal survival. Our data suggest that modulation of MICU1 and MCU may be a promising target for future neuroprotective strategies in PINK1‐related PD.

## Materials and methods

### Animal maintenance

Adult *wt* (AB and TL *wt* lines) and *pink1* ^*−*/*−*^ (Flinn *et al*., 2013) zebrafish were maintained according to methods previously described (Matthews *et al*., 2002). Adult zebrafish and larvae were kept in E3 medium at 28.5 °C.

### RT‐PCR

RNA was isolated from a pool of 20 embryos using Tri reagent (Sigma Aldrich) and cDNA was generated using Verso cDNA synthesis kit (Thermo Scientific). RT‐primers (5′‐AGACTGTCAGGAGAGCACAC‐3′, 5′‐GACGTACAGAAATCACCGGC‐3′) were designed against *mcu* (ENSDART00000130884) and RT‐PCR was performed to determine *mcu* expression at different developmental stages (1, 24, 48 and 72 hpf). Primers (5′‐GGCAAATCTGCCAGA GACAT‐3′, 5′‐TGTCCGTGTTCCACTTCTCA‐3′) were also designed against the transcript *vdac1* (ENSDART00000066373) and RT‐PCR was performed to determine *vdac1* expression during early developmental stages (1, 24, 48 and 72 hpf). Each PCR reaction mixture consisted of 10 μL Biomix Red TM (Bioline), 2 μL forward primer, 2 μL reverse primer, 4 μL H_2_O and 2 μL of cDNA. The reaction was heated to 95 °C for 3 min with 34 cycles (95 °C for 30 s, 60 °C for 30 s, and 72 °C for 1 min). This was followed by a final incubation at 72 °C for 5 min.

### Morpholino injections

Morpholino (Gene Tools) were employed to functionally knockdown *mcu*,* vdac1* and *micu1*. Single cell stage embryos were selected to microinject 1 nL of morpholinos directed against *mcu* (0.9 mm of *mcu* e2i2 morpholino (MO) – 5′‐CATCAAGAGTAAAGCACTAACCTGG‐3′), *vdac1* (0.9 mm of *vdac1* e5i5 MO – 5′‐TGCATAAATTCGTACCCAGTGTTTG‐3′ combined with *vdac1* i6e7 MO – 5′‐CCGTCATTCCTGATATTAAACACA‐3′) and *micu1* (0.5 mm of *micu1* e10i10 MO – 5′‐GACTCTTATCAAGCTGCCCACCTGA‐3′). The efficacy of the morpholino‐mediated knockdown was assayed by RT‐PCR using primers against flanking exons of the MO target site. The primer sequences are as follows: *mcu*‐ F 5′‐AGAGATGGCTGCGAAAGTGT‐3′: R 5′‐AAAACACCCACCGTGTCACT‐3′; *vdac1‐* F 5′‐GCTGAACACGGCCTGACCTT‐3′: R 5′‐CCT CCGGCGTTAATGTTCTT‐3′: *micu1‐* F 5′‐GGCTGATCTGAAGGGCAAAC‐3′: R 5′‐GTCCGACAGCTCCACTTTTG‐3′.

### Quantitative‐PCR

Twenty embryos were used per genotype for RNA isolation. Quantitative PCR (Q‐PCR) analysis was conducted with gene‐specific primers (*mcu*‐ 5′‐AGACTGTCAGGAGAGCACAC‐3′, 5′‐GACGTACAGAAATCACCGGC‐3′; *emre*‐ 5′‐TGTGTTCTCCTCAGCTCGG‐3′, 5′‐CTGATCAGAGTGCCGACGTA‐3′; *mcur1*‐ 5′‐CTCATGCAATGGTCCAGCTG‐3′, 5′‐CTGCTGTGTCTTGGTAGCCA‐3′; *micu2*‐ 5′‐TGAAGAGGACAACGAGGCAT‐3′, 5′‐ACAGAGATGGCGAAGTCCTC‐3′; *micu1*‐ 5′‐GGCGGGCTCTTCAGTTAAAC‐3′, 5′‐CATACTCCATCACCTTGCGG‐3′), which were optimized for annealing temperature, concentration and efficiency. SYBR green master mix (Agilent) was utilized for the enzyme reaction, reaction incubated and analyzed by Stratagene MxPro 3000p Real Time PCR machine (Stratagene). The fold change in expression was quantified by normalizing the threshold cycle (CT) values of the target mRNAs to the CT values of the internal control *ef1alpha* (ENSDARG00000020850) in the same samples (∆CT = CT_Target_ − CT_EF1alpha_). It was further normalized with the *wt* control (∆∆CT = ∆CT − CT_Control_). The fold change in expression was then obtained by 2^−∆∆CT^. The results are representative of three biological replicates, each consisting of three technical replicates.

### Chemical treatment

The pharmacological inhibitor of MCU, Ruthenium red (RR) was used to confirm the results from the functional knockdown of *mcu* using MO. RR (Sigma Aldrich) was dissolved in sterile water at 10 μm concentration and treated to larvae in E3 medium for 3 days (*n* = 10 of embryos per genotype and experiment).

### Whole mount *in situ* hybridization

Whole mount *in situ* hybridization (WISH) was performed as previously described (Jowett & Lettice, 1994; Thisse & Thisse, 2008; Shimizu *et al*., 2015). Specific primers with artificially introduced T7 and SP6 polymerase sequence were used to perform PCR amplification of desired gene of interest. The primer sequences are as follows: *th‐* F 5′‐AATTAACCCTCACTAAAGGGAGAATGCCGAATTCAAGCAGCTCCAC‐3′: R 5′‐TAATACGACTCACTATAGGGAGAAGCGTGCCGTATGTACTGTGTGC‐3′; *mcu‐* F 5′‐TAATACGACTCACTATAGGGGCTGAGTAAGAAAGCCGAGC‐3′: R 5′‐GATTTAGGTACTATAGGCACCACATCCCGAAATCTC‐3′; *micu1‐* F 5′‐TAATACGACTCACTATAGGGAGACATCAATGAACACAGGGACG‐3′: R 5′‐ATTTAGGTGACACTATAGAAGATCACACAACATGGTCCGACA‐3′; *vdac1*‐ F 5′‐TAATACGACTCACTATAGGGTCGACTCCTCCTTTTCTCCA‐3′: R 5′‐GATTTAGGTGACACTAATAGACGGACGCAGCAGGTAGTAT‐3′. The PCR product was used as a template for synthesis of the digoxigenin‐labeled antisense RNA probe using *in vitro* transcription. Specifically, an RNA probe against tyrosine hydroxylase (TH) was synthesized to label dopaminergic neurons. An anti‐DIG antibody coupled to alkaline phosphatase was used to detect the hybridized RNA probe. Zebrafish larvae were embedded in 3% methylcellulose and photographed. The mean number of these diencephalic dopaminergic neurons in experimental larvae were calculated over three independent experiments (*n* = 10 of embryos per genotype and experiment).

### Mitochondrial complex I–IV assays

Assays were performed as previously described (Flinn *et al*., 2013). Briefly, 5 dpf zebrafish larvae were harvested into Eppendorf tubes of 20 larvae per experimental condition, and the mitochondrial fraction was isolated. Homogenates were freeze thawed three times in liquid N2 before being used for spectrophotometric assessment of respiratory chain complexes. The specific activity of complex I–IV was normalized to that of citrate synthase. Complex I activity is determined by following the oxidation of NADH to NAD+ and the simultaneous reduction of a dye which leads to increased absorbance at OD = 450 nm. All mitochondrial complex activity assays were performed in triplicate.

### Data analysis

In all experiments, error bars indicate the standard error of mean (SEM, at least three independent experiments). All experiments were carried out in biological triplicate unless specifically stated otherwise. The following statistical tests were applied to determine statistical significance: Q‐PCR: unpaired two‐tailed, two sample *T* test; TH *in situ* hybridisation experiments (counting of dopaminergic neurons) and complex I activity assay experiments: One way anova followed by Tukey's multiple comparison test.

### *In silico* modeling

To integrate the experimental data mechanistically, we adapted our previous developed model (Komin *et al*., 2015). The rate equation model takes into account the cytosolic Ca^2+^ dynamics that is mainly driven by Ca^2+^ release from the endoplasmic reticulum (ER) through inositol trisphosphate receptors (IP3Rs) into the cytosol and removal of cytosolic Ca^2+^ back into the ER by sacro‐ER Ca^2+^ ATPases (SERCAs) and into the extracellular space by plasma membrane Ca^2+^ ATPases (PMCAs) (Fig. 4A). Within the cytosol Ca^2+^ is taken up by mitochondria via the MCU where it triggers ATP production by TCA cycle activity and released into the cytosol by the sodium Ca^2+^ exchanger (NCX). The adapted model considers the corresponding Ca^2+^ fluxes and the triggered mitochondrial activity is simulated by a first principle‐based module (Bertram *et al*., 2006). The resulting cytosolic ATP concentration subsequently determines SERCA and PMCA mediated fluxes and therefore cytosolic Ca^2+^ dynamics. The mathematical equations were implemented in a customized Ca^2+^ program.

## Results

### MCU inactivation rescues dopaminergic neurons in PINK1 deficiency

**Figure 1:**
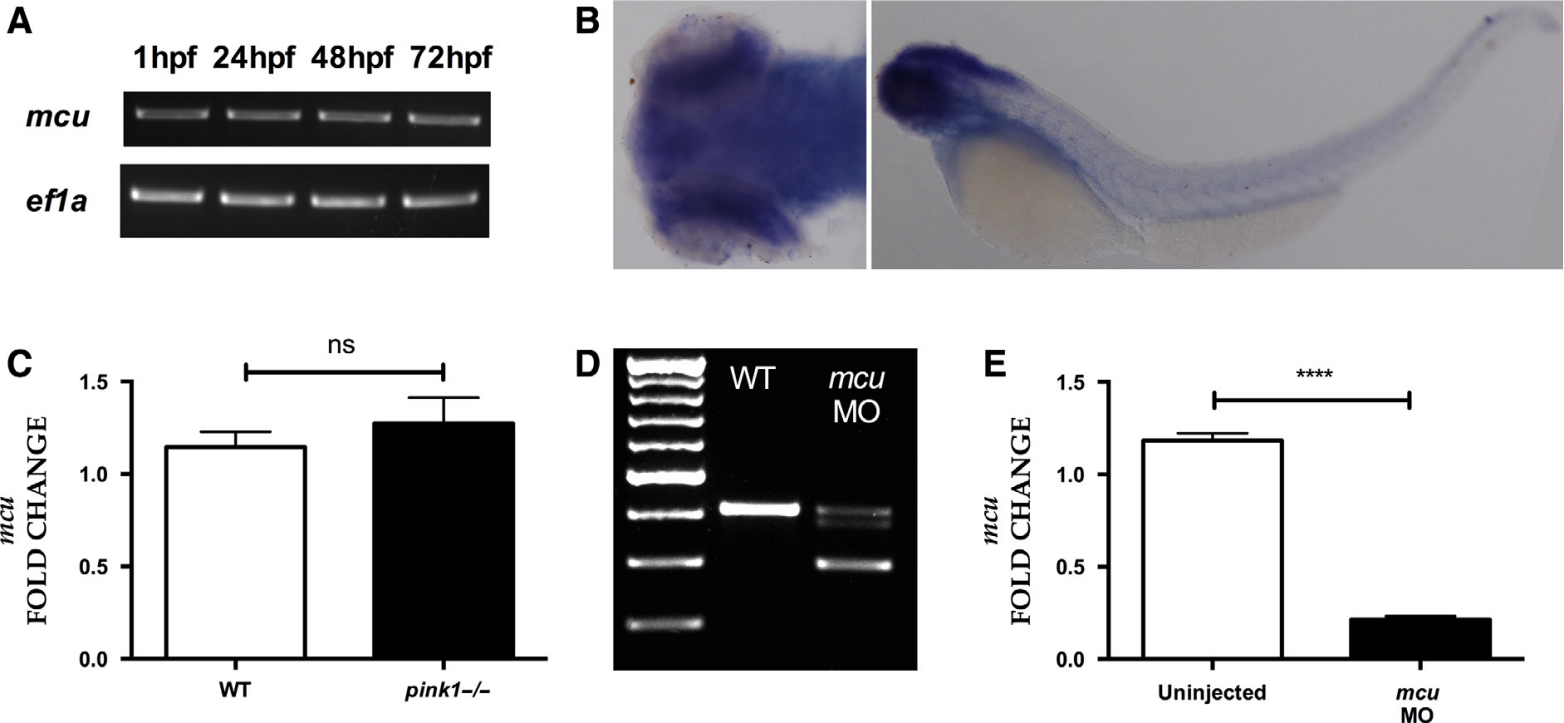
*mcu* expression. (A) RT‐PCR analysis showing consistent expression of *mcu* throughout development (1, 24, 48 and 72 hpf). (B) *In situ* hybridization with dorsal (left) and lateral (right) views of *mcu* expression in 3 dpf zebrafish larvae, demonstrating strong expression in the brain. (C) Quantification of *mcu* mRNA at 3 dpf, demonstrating similar expression levels in *wt* and *pink1* ^−/−^. (D) RT‐PCR analysis of *mcu* knockdown using antisense morpholino (MO) at 3 dpf, demonstrating the marked effect of MO mediated *mcu* k/d on *mcu* wild type transcript levels. (E) Q‐PCR based confirmation of marked effect of MO‐mediated *mcu* k/d on *mcu* mRNA transcript levels (****P < 0.0001). [Colour figure can be viewed at wileyonlinelibrary.com].

**Figure 2:**
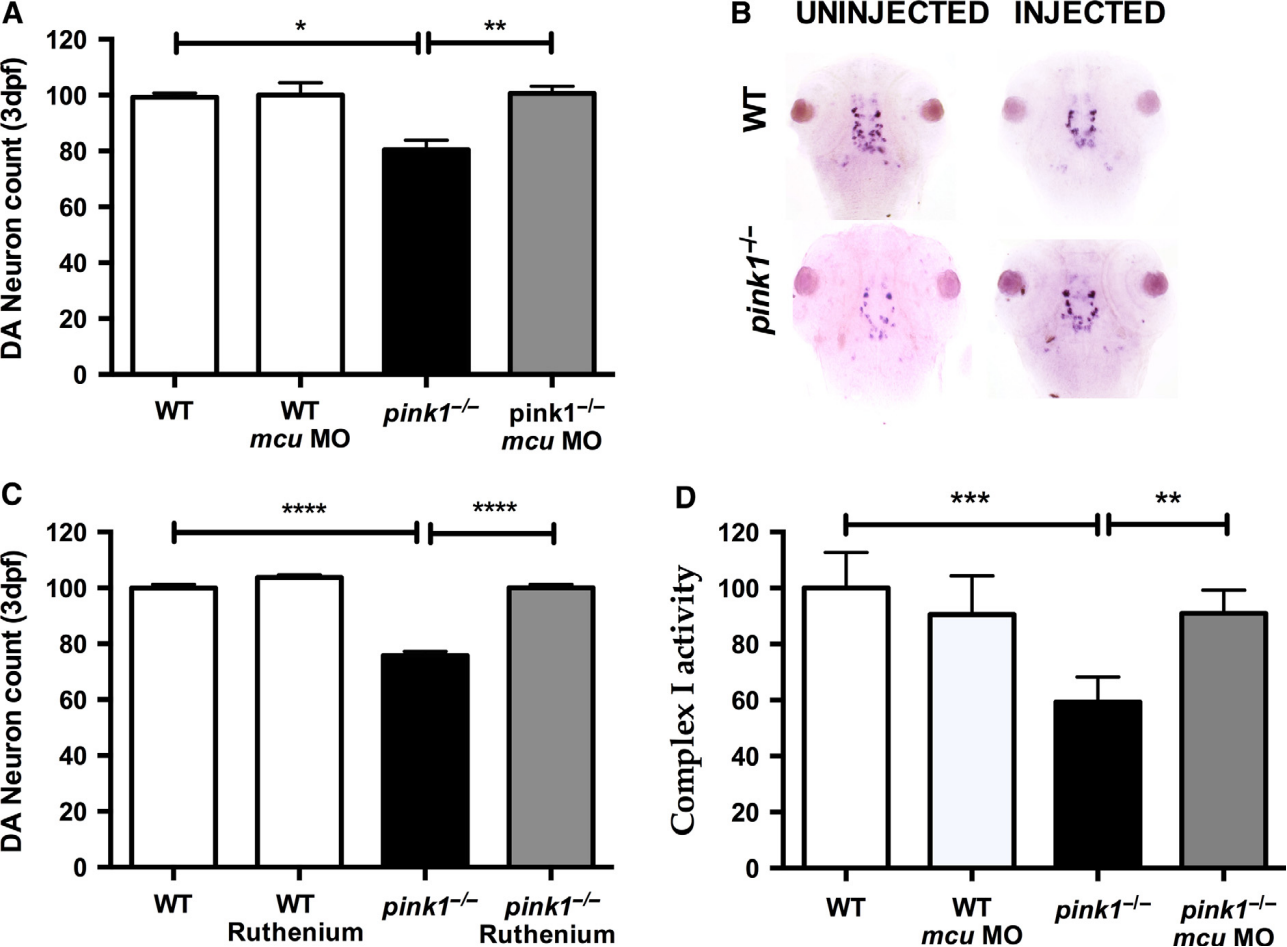
Knockdown of *mcu* leads to rescue of dopaminergic neurons. (A) Dopaminergic (DA) neuronal cell count in *wt*,* wt* MCU (*wt* microinjected with MO against *mcu*), *pink1* ^−/−^, *pink1* ^−/−^ MCU (*pink*1^−/−^ microinjected with MO against *mcu*) zebrafish larvae at 3 dpf. DA neuronal cell count is reduced in *pink1* ^*−*/*−*^ (*P = 0.012) but completely rescued after MCU inactivation (**P = 0.0085). The scale on the *y* axis reflects % of DA neurons compared to *wt* controls. (B) Representative image showing dopaminergic neurons in the diencephalon [using tyrosine hydroxylase (TH) whole mount *in situ* (WISH) staining] in *wt* controls and *pink*1^−/−^ zebrafish injected with and without *mcu* MO. (C) Dopaminergic (DA) neuronal cell count in *wt*,* wt* embryos treated with Ruthenium red (RR) (*wt* RR), *pink*1^−/−^ and *pink*1^−/−^ embryos treated with RR (*pink*1^−/−^ RR) with complete rescue of DA neurons in *pink*1^−/−^ larvae after RR treatment (****P < 0.0001). The scale on the *y* axis reflects % of DA neurons compared to *wt* controls. (D) WT vs *pink1* ^−/−^ (***P = 0.0007). Mitochondrial complex I activity is restored in 5 dpf *pink*1^−/−^ following *mcu* k/d (**P = 0.0071). Complex I activity is measured in μmol oxidized NADH per 1 unit of citrate synthase activity and set to 100% for the complex I activity in WT controls. The scale on the y axis reflects % of complex I activity compared to *wt* controls. [Colour figure can be viewed at wileyonlinelibrary.com].

*mcu* (ENSDARG00000101175), the zebrafish orthologue of human *MCU* (ENSG00000156026), was identified using Ensembl genome browser. *mcu* shares 70.7% DNA and 78.8% protein identity with human MCU. *mcu* was already expressed at 1 h post fertilization (hpf), expression was then constant throughout development (Fig. 1A). WISH of *mcu* indicated predominant expression in brain tissue (Fig. 1B). Q‐PCR analysis revealed similar *mcu* mRNA transcript levels in *pink1* ^*−*/*−*^ and wild type (*wt*) larvae at 3 dpf (Fig. 1C). We next applied the morpholino (MO) antisense strategy to genetically inactivate *mcu*. Injection of a splice site MO targeted at the exon 2/intron 2 junction of *mcu* at single cell stage resulted in a dose‐dependent skipping of exon 2 (Fig. 1D) with marked reduction in mRNA transcript levels (Fig. 1E). This MO‐induced knockdown (k/d) of *mcu* did not alter the overt development, spontaneous motor behavior or phenotypic characteristics until 5 dpf (data not shown). As previously observed, the number of diencephalic dopaminergic neurons within the 1, 2, 4 and 5 subgroups according to the Rink‐Wullimann classification were reduced in *pink1* ^*−*/*−*^ at 3 dpf (*P* = 0.0128) (Rink & Wullimann, 2001; Flinn *et al*., 2013). At 3 dpf, *mcu* k/d did not change the number of dopaminergic neurons in *wt* larvae, but resulted in a marked rescue of dopaminergic neurons in *pink1* ^*−*/*−*^ (*P* = 0.0085) (Fig. 2A,B). We next sought to validate the observed protective effect of genetic MCU inactivation using a different, pharmacological approach. RR has long been recognized as a pharmacological inhibitor of mitochondrial calcium influx prior to the discovery of the MCU as such (Moore, 1971). Treatment of *wt* larvae with RR at a concentration of 10 μm for 3 days did not have an effect on the number of diencephalic dopaminergic neurons, but – as predicted – had a marked protective effect on the seven dopaminergic neurons in *pink1* ^*−*/*−*^ larvae (*P* < 0.0001, Fig. 2C), similar to the rescue effect observed after genetic MCU inactivation. We next sought to identify the mechanism underlying the observed rescue effect of MCU inactivation in PINK1 deficiency. As mentioned earlier, we had previously observed a marked decrease in the activity of complex I in *pink1* ^*−*/*−*^ (Flinn *et al*., 2013). We hypothesized that MCU inactivation rescues dopaminergic neurons via normalization of mitochondrial respiratory chain function. As predicted, MO‐mediated genetic inactivation of MCU as described above had a rescue effect on complex I activity in *pink1* ^*−*/*−*^ larvae at 5 dpf (*P* = 0.007, Fig. 2D).

### The effect of VDAC1 and MCU inactivation are distinct

**Figure 3:**
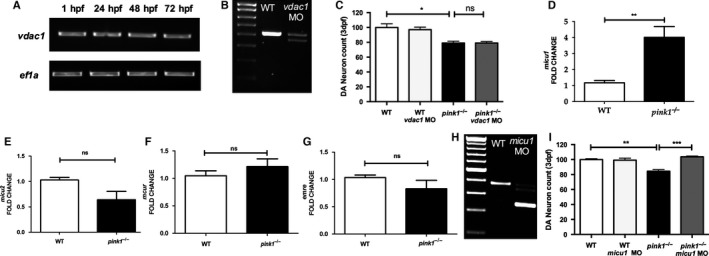
VDAC1 and MICU1 analysis. (A) RT‐PCR analysis demonstrating expression of vdac1 at 1, 24, 48 and 72 hpf. (B) RT‐PCR analysis of vdac1 in *wt* controls and after MO mediated *vdac1* k/d, demonstrating a marked effect of the *vdac1* MO on *vdac1 wt* transcript levels. (C) Dopaminergic (DA) neuronal cell count in *wt*,* wt vdac1* (*wt* injected with MO against *vdac1*), *pink*1^−/−^, *pink*1^−/−^ *vdac1* (*pink*1^−/−^ injected with MO against *vdac1*) at 3 dpf, reflecting the lack of an effect of VDAC1 inactivation on DA neuronal cell count in *pink*1^−/−^ (*P* > 0.99) (*P = 0.0105). (D) qPCR analysis of *micu1*, showing significant upregulation of *micu1* in *pink*1^−/−^ larvae at 3 dpf compared to *wt* (**P = 0.0066). (E) qPCR analysis of *micu2*, showing a non‐significant down regulation of *micu2* in *pink*1^−/−^ larvae at 3 dpf compared to *wt* (^ns^ *P* = 0.0902). (F) qPCR analysis of *mcur*, showing a non‐significant upregulation of *mcur* in *pink*1^−/−^ larvae at 3 dpf compared to *wt* (^ns^ *P* = 0.2088). (G) qPCR analysis of *emre*, showing a non‐significant down regulation of *emre* in *pink*1^−/−^ larvae at 3 dpf compared to *wt* (^ns^ *P* = 0.2712). (H) RT‐PCR analysis of *micu1* at 3 dpf in uninjected larvae (WT) and after MO mediated k/d (*micu* MO), demonstrating the marked effect of *micu1* MO injection on *micu1* mRNA transcript levels. (I) Dopaminergic (DA) neuronal cell count in *wt*,* wt micu1* (*wt* injected with MO against *micu1*), *pink1* ^*−*/*−*^, and *pink1* ^*−*/*−*^ *micu1* (*pink*1^−/−^ injected with MO against *micu1*) zebrafish larvae at 3 dpf, demonstrating the rescue effect of MICU1 inactivation on DA neurons in PINK1 deficiency (***P = 0.0004).

We next investigated whether the observed rescue effect of either genetic or pharmacological MCU inhibition on dopaminergic neurons in PINK1 deficiency is specific or could also be observed after inhibition of the outer mitochondrial membrane channel VDAC1. *vdac1* (ENSDARG00000045132) the zebrafish orthologue of human *VDAC1* (ENSG00000213585) shares 77.1% DNA sequence identity with *VDAC1* and 85.5% protein identity. *vdac1* is already expressed at 1 hpf with similar expression levels at 1, 2 and 3 dpf (Fig. 3A). Marked MO‐mediated knockdown of *vdac1* was achieved using a combination of two splice site MOs directed against the exon 5/intron 5 splice site and the intron 6/exon 7 (Fig. 3B). In contrast to MCU inactivation, MO‐mediated k/d of *vdac1* did not rescue diencephalic dopaminergic neurons in *pink1* ^*−*/*−*^ larvae (*P* > 0.99, Fig. 3C).

### Altered functional regulation of MCU in PINK1 deficiency

As described above, *mcu* expression levels in *pink1* ^*−*/*−*^ larvae were similar to the expression levels observed in *wt* larvae but the marked rescue effect of *mcu* silencing suggested a possible dysregulation in MCU function. We hypothesized that this dysregulation may be due to altered expression levels of the MCU regulators *micu1* (ENSDARG00000063358), *micu2* (ENSDARG00000009939), *emre* (ENSDARG00000095826) or *mcur* (ENSDARG00000016964) in *pink1* ^−/−^. We observed a 4 fold upregulation (4.01, SD ± 1.36, Fig. 3D) of *micu1* (*P* = 0.0066) in *pink1* ^−/−^ compared to *wt* larvae at 3 dpf. mRNA expression levels of *micu2*,* emre* and *mcur* also varied between *pink1* ^−/−^ and *wt* larvae but the observed differences did not reach statistical significance (Fig. 3E–G). *micu1* (ENSDARG00000063358) the zebrafish orthologue of human *MICU1* (ENSG00000107745) shares 91.38% DNA identity and 74.3% protein identity with *MICU1*. We next applied the MO k/d strategy to further investigate the possible functional relevance of the observed *micu1* transcriptional upregulation which suggested a possible change in the calcium trafficking properties of MCU. Splice site MO directed against the exon 10/intron 10 boundary established k/d of *micu1* (Fig. 3H). MICU1 deficient zebrafish larvae did not develop any overt developmental, behavioural or phenotypical abnormalities (data not shown). MO‐mediated knockdown of *micu1* resulted in protection of the diencephalic dopaminergic neurons similar to the effect observed after *mcu* k/d (*P* = 0.0004; Fig. 3I). Taken together, our data suggest MICU1 mediated activation of MCU in PINK1 deficiency.

### *In silico* modeling

**Figure 4:**
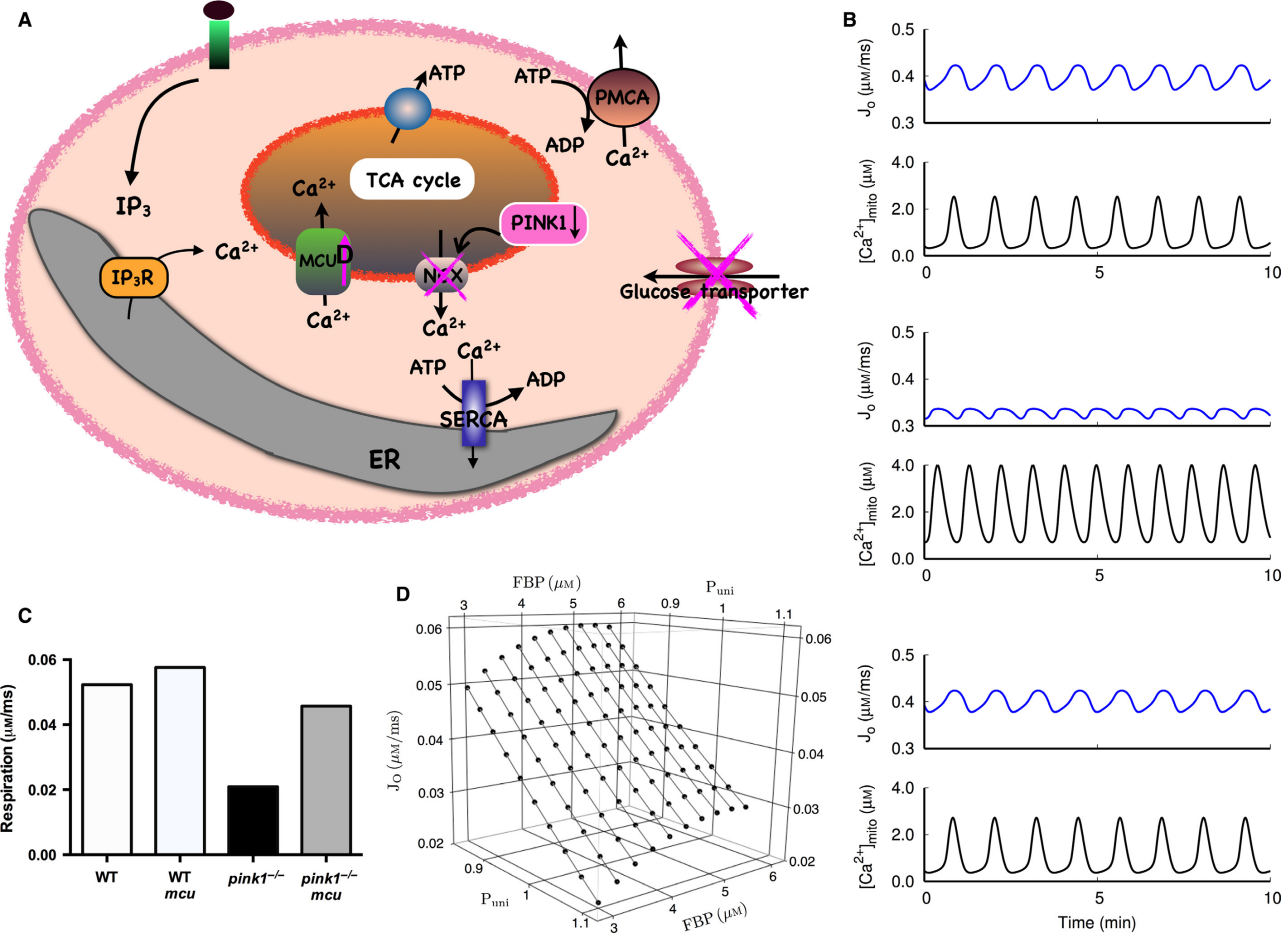
Mathematical modeling of Ca^2+^ triggered mitochondrial energy. (A) Scheme of the rate equation model and the considered effect of PINK1 deletion. The model combines cytosolic Ca^2+^ dynamics driven by IP_3_R mediated release from the endoplasmic reticulum (ER) and removal by sarco/endoplasmic reticulum Ca^2+^‐ATPase (SERCA) and plasma membrane Ca^2+^ ATPase (PMCA) pumps with the induced mitochondrial ATP production. The effect of PINK1 deficiency on glucose uptake and mitochondrial Ca^2+^ handling is indicated in purple. NCX = sodium Ca^2+^ exchanger. (B) Dynamic modeling results of mitochondrial Ca^2+^ concentrations and resulting oxygen flux (Jo) for *wt*,* pink1* ^*−*/*−*^ and *pink1* ^*−*/*−*^ + MCU down regulation conditions. Compared to the *wt* scenario (top panel), *pink1* ^*−*/−^ leads to mitochondrial Ca^2+^ overload and subsequently to smaller Jo amplitudes indicating decreased respiration (middle panel). This effect could be compensated by down regulation of MCU reestablishing mitochondrial Ca^2+^ homeostasis and mitochondrial activity (bottom panel). (C) Respiration activity for the different conditions. The oxygen consumption rate as an indicator for respiratory activity exhibit a similar pattern to the dopaminergic neuronal survival and complex I activity (see Fig 2). (D) Intensive parameter scans have shown the stability of the compensatory effect of MCU down regulation compared to *wt* conditions (*P*_uni_ = 1) on respiration measured by oxygen consumption. The model also suggests a potential intervention by up regulation of glucose uptake leading to higher fructose biphosphate (FBP) concentrations. [Colour figure can be viewed at wileyonlinelibrary.com].

To investigate the potential role of energy metabolism as a mechanism for the observed changes in dopaminergic neuronal viability based on the selective vulnerability hypothesis (Chan *et al*., 2009), we simulated the different scenarios with our recently developed and validated *in silico* model (Komin *et al*., 2015). The rate equation model considers the cytosolic dynamics and its impact on mitochondrial activity based on fluxes between the two compartments mediated by well‐established molecular interactions summarized in in Fig. 4A. Based on our previously determined parameter set describing *wt* dynamics (Fig. 4B), the mechanistic approach allows for testing the effect of PINK1 loss of function (Gandhi *et al*., 2009) and the impact of MCU activity (Fig. 4A). To simulate the impaired PINK1 case, we increased the flux through the MCU by 10% and decreased the flux rate of the Na/Ca^2+^ exchanger by 10% as well as the glucose uptake rate by 30%. These values were estimated by scanning the model parameters in the physiological range in order to reproduce the experimental results. While the resulting cytosolic Ca^2+^ dynamics (Ca_*c*_) is hardly modified compared to *wt* condition (upper panel Fig. 4B), the mitochondrial Ca^2+^ concentration (Ca_*m*_) and oxygen flux (*J*_*0*_) exhibit a significant difference (middle panel Fig. 4B). In particular, the amplitude of *J*_*0*_ that describes the oxygen consumption rate and is therefore a measure of respiration activity, decreases dramatically due to mitochondrial Ca^2+^ overload. This effect can be compensated in the model by decreasing MCU activity by 14% preventing mitochondrial Ca^2+^ overload and restoring respiratory (lower panel Fig. 4B). The different scenarios are summarized in Fig. 4C with respect to the oxygen consumption rate corresponding to respiratory activity. The described flux modifications lead to a very similar picture as in the experimentally determined neuronal survival. Since neuronal survival will not depend linearly on respiration activity, we performed comprehensive model parameter scans to test the stability of our results. Fig. 4D exhibits a parameter scan with respect to MCU activity (*P*_uni_) relative to *wt* conditions (*P*_uni* *_= 1) and fructose‐bisphosphate (FBP) that 10 depends on the glucose uptake rate. The obtained dependency of respiration on *P*_uni_ and FBP as well as similar results from additional parameter scans demonstrate that the mechanistic modeling support of the experimental findings do not depend on specific parameter combinations but are robust for a wide physiological parameter range.

## Discussion

Calcium has repeatedly been implicated in the pathogenesis of PD. We now provide further evidence for a complex interplay between altered mitochondrial function, impaired mitochondrial calcium homeostasis and the MCU complex in PINK1 deficiency. Our observation of normal development in MCU‐deficient zebrafish larvae is in keeping with the normal development of *MCU* ^−/−^ mice on the outbred, mixed CD1 genetic background (Murphy *et al*., 2014). Differences in the genetic background may explain the discrepancy between the lack of an effect of MCU inactivation on zebrafish development in our experiments compared to the marked effect observed by others (Prudent *et al*., 2013). There are ongoing concerns about potentially misleading conclusions resulting from MO‐based experiments. However, erroneous effects of MO are typically due to nonspecific toxic effects (Kok *et al*., 2015; Stainier *et al*., 2015). None of the typical morphological characteristics of these often p53‐mediated, non‐specific off‐target effects were observed after MO‐mediated k/d of *mcu*. Furthermore, we were able to validate our MO‐based data using a completely different strategy, namely pharmacological inhibition of MCU with RR. We therefore consider it to be unlikely that the observed rescue effect of *mcu* k/d could be due to a non‐specific off‐target effect of the MOs used in our experiments. Further validation of the presented data could be undertaken in double‐knockout zebrafish lines which are both *pink1* and *mcu* mutant to further reduce the already unlikely possibility of the observed rescue effect being due to a non‐specific off‐target effect. MCU k/d had previously been shown to reduce excitotoxicity‐induced neuronal cell death after NMDA receptor activation *in vitro* (Qiu *et al*., 2013). However, excitotoxic stress is not considered to be a key player in PD pathogenesis and modulation of excitotoxic stress is unlikely to be the underlying mechanism in our zebrafish model of PINK1 deficiency.

MICU1 is thought to have a major role in regulating MCU channel activity during high cytosolic calcium concentrations (Ahuja & Muallem, 2014). The specific increase in *micu1* expression as well as the protective effect of *micu1* k/d on dopaminergic neurons suggests that MICU1 activation is involved in the mechanisms leading to dopaminergic neuronal cell loss via mitochondrial calcium overload in PINK1 deficiency. Further studies in other *in vitro* and *in vivo* model systems of PINK1 deficiency as well as, ideally, assessment in *PINK1* mutant patient tissue is necessary to determine whether the observed transcriptional upregulation of *micu1* is specific for *pink1* ^−/−^ zebrafish or more generally observed in PINK1 deficiency. However, any attempts to further study the observed rescue effect of both MCU and MICU1 inactivation in higher vertebrates will be challenging due to the absence of dopaminergic neuronal cell loss in *Pink1* k/o mice (Oliveras‐Salva *et al*., 2011). Of note, lack of MCU does not protect heart cells against ischemic cell death despite an abolished effect of ischemia on the activation of the mitochondrial permeability transition pore in *Mcu* ^−/−^ mice (Pan *et al*., 2013). Further work is required to confirm that the observed beneficial effect of *mcu* k/d in our *pink1* ^*−*/*−*^ larvae is indeed due to a modulatory effect on mitochondrial calcium homeostasis with resulting normalization of mitochondrial respiratory chain function rather than exerting its effect via modulation of other mechanisms linked to the pathogenesis of PD such as an effect on mitophagy (Sousa *et al*., 2003; Deas *et al*., 2011).

All three VDAC isoforms are equivalent in allowing mitochondrial calcium loading upon agonist stimulation, but only VDAC1 silencing selectively impairs the transfer of the low‐amplitude apoptotic calcium signals (De Stefani *et al*., 2011). VDAC1 has been reported to physically interact with MCU (Liao *et al*., 2015). VDAC1 has been implicated in neuronal cell death in both Alzheimer's disease (AD) and PD (Reddy, 2013; Alberio *et al*., 2014). In addition to its role in Calcium homeostasis, VDAC1 has also been implicated in PINK1/Parkin‐mediated mitophagy (Geisler *et al*., 2010). We were therefore intrigued to observe a complete lack of a neuroprotective effect of VDAC1 inactivation in *pink1* ^−/−^ zebrafish. This further suggests a specific and prominent role of MCU in PINK1 deficiency‐linked neuronal cell death.

Due to the role of Ca^2+^ in activation of mitochondrial respiration (Chan *et al*., 2009), our findings point to energy metabolism as a contributing effector of neuron survival what is further supported by our mechanistic *in silico* studies. This perspective is in accordance with the selective vulnerability hypothesis that assumes the larger energetic load of dopaminergic neurons to be a major reason for their increased cell death rate in PD (Pissadaki & Bolam, 2013).

## Author contributions

SS, MK MDC, HM, SS and MB undertook experiments and analysed the data; MM and AS developed the *in silico* model, OB and JK developed the underlying hypothesis and supervised the project. All authors contributed to the writing of the manuscript.

## Conflict of interests

The authors declare no conflict of interest.
